# Depression, memory and electroconvulsive therapy

**DOI:** 10.1192/bjb.2018.59

**Published:** 2019-04

**Authors:** Rudi Coetzer

**Affiliations:** 1 North Wales Brain Injury Service, Betsi Cadwaladr University Health Board NHS Wales, UK; 2 School of Psychology, Bangor University, UK

## Abstract

Current neuroscience suggests that although short-term memory difficulties frequently occur immediately after electroconvulsive therapy (ECT), longer-term problems are less common. However, gaps in our knowledge remain regarding longer-term cognitive problems after ECT, including memory function. Some of these relate to the complexities surrounding cognitive testing and interpretation of test results. An important question in clinical decision-making is why, despite current evidence suggesting long-term memory problems are less frequent, some patients still report subjective memory difficulties. To further advance clinical practice and the neuroscience surrounding post-ECT cognitive function, assessment of cognitive function, possibly including neuropsychological testing, should potentially become more routine as part of clinical practice.

**Declaration of interest:**

None

Measuring treatment response after depression is often not entirely straightforward in clinical practice. What matters to clinicians might not always perfectly align with what is important to their patients. For example, the DSM-5 lists a combination of physical, cognitive, affective and behavioural diagnostic criteria representing major depressive disorder (MDD).^1^ For patients with MDD, subjectively, fatigue is sometimes the most disconcerting symptom, whereas for others it might be impaired concentration or the persistent feelings of sadness and hopelessness, resulting in a range of subtly different presentations. Similarly, a wide range of effective treatments for MDD are now available, depending on patients' clinical presentation. Treatments range from pharmacological approaches, talking therapies and physical exercise, to electroconvulsive therapy (ECT) or a combination of these, among others. Treatment approaches all have different levels of efficacy, potential side-effects, and accordingly their acceptability to patients with MDD varies.

A recent retrospective study found that patients were generally satisfied with ECT as a safe and effective treatment for depression.^2^ Nevertheless, one of the suggested adverse side-effects of ECT concerns possible memory problems. Whether ECT can have a deleterious effect on memory presents an important question for both the clinician and patient when discussing treatment options. An appraisal of the current neuroscience knowledge on memory and ECT can provide some guidance to further inform clinical decision-making, including addressing patients' concerns regarding treatment choice. Lezak *et al*, reviewing neuropsychology and other neuroscience findings concerning ECT and memory, conclude that memory difficulties immediately post-ECT can be present in some patients, but that it is less common beyond this period, even in patients who have had longer-term treatment.^3^ Similarly, Kirov *et al* conclude that long-term memory effects are rare and that ECT appears to be safe as regards adverse neuropsychological outcomes.^4^ Furthermore, a recently published, large cohort study found that the risk of developing dementia in patients previously treated with ECT was not significant.^5^ These findings might reassure clinicians, but what about patients' views? Understanding patients' preferences and concerns as regards treatment choice is an important aspect of clinical decision-making and ultimately for determining overall treatment response.

## Memory complaints post-ECT

Vann Jones and McCollum^6^ report the findings from their systematic review of ECT and subjective memory complaints. Although there were methodological limitations to some of the included studies, which made definitive conclusions difficult, their review does suggest that generally subjective memory ratings improve over time, and that ultrabrief-pulse ECT potentially has less side-effects. Interestingly, another study not included in their review reported that although cognitive function appears to be largely unchanged in many (but not all) areas of neuropsychological functioning, improved depression long-term post-ECT appears to be associated with improvement in some cognitive functions.^7^ Vann Jones and McCollum's review raises some interesting questions for clinical practice and neuroscience. In particular, if the above (and other) findings from the research literature is correct in concluding that ECT is safe as regards memory functions, why is it that a number of patients consistently continue to report longer-term subjective memory difficulties after ECT?

Addressing the above question is potentially of considerable relevance not only to patients, but also clinicians. For example, from a policy and practice perspective there is an acknowledgement of the importance of considering cognition as part of ECT service delivery. The Royal College of Psychiatrists Statement on Electroconvulsive Therapy advises that cognitive functioning should be assessed before ECT and monitored after every three or four ECTs, and also after the final ECT.^8^ Furthermore, the Statement indicates that in addition to orientation being assessed after each ECT, retrograde amnesia, new learning and subjective memory complaints should be tested more than 24 h post-ECT. However, exactly how to perform testing of these functions is not specified. Interestingly, looking at some current National Health Service ECT policies fails to identify a consistent and clearly defined approach to assessing cognition in ECT services. To summarise, although the relevance of assessing cognition after ECT is clearly acknowledged, how to do this remains less clear. At present, there is not a straightforward answer doctors can give to patients receiving ECT, if they ask exactly how their memory functions will be monitored.

## Assessing cognition post-ECT

Assessment of cognition can be performed at different levels, providing objective or subjective data. Patients can be asked about their own views on their cognitive functioning, which provide subjective data. Objective testing can be performed at the bedside, or can constitute formal neuropsychological testing. In the latter case, the patient's results are compared against standardised norms as well as their own predicted premorbid level of functioning. Patient effort is also measured to identify any underperformance that might influence the interpretation of test results. Cognitive domains assessed generally include information processing, language functions, new learning and retention, construction, visual–spatial abilities and executive control functions, among several others. Kolar suggests that neuropsychological testing should be an integral part of ECT service provision.^9^ In ECT services, testing will most likely focus on cognitive domains such as new learning and retention, as well as information processing. Although the assessment of cognitive functions appears straightforward enough, interpretation of the results is not. This may be one of the reasons why wider implementation of cognitive assessment post-ECT has not occurred.

Returning to the relevance of diagnostic criteria as outlined at the beginning of this editorial provides a good example of some of the complexities surrounding post-ECT cognitive assessment. Cognitive symptoms of MDD, such as poor concentration, can be affected by some of the other features of the disorder. For example, insomnia can adversely affect concentration, which will have a negative downstream effect on memory (new learning and retention). The same applies to fatigue. In terms of the statistical interpretation of neuropsychological test results, one example would be that consideration should be given to the possibility that when compared with standardised norms, depressed patients may already (before ECT) be underperforming, or even be near floor level (a score that is too low to reliably identify any further decline in performance). This is one of the reasons why the individual patient's neuropsychological profile ideally should be interpreted against their own predicted premorbid level of cognitive function by using, for example, demographic data or formal tests of premorbid intellectual function. Other issues related to interpretation of test results concerns for example determining what constitutes a reliable clinical change during serial testing, or identifying what represents an impairment versus a below average performance of a given cognitive function in a test protocol.

There are also non-cognitive factors to consider when interpreting data. Particularly relevant to Vann Jones and McCollum's study would be the effect of patients' self-awareness (or ‘insight’) on their subjective judgements of memory function. Problems in this area can influence how patients rate their memory performance and result in very different scores from actual objective test performance. For example, a small study looking at repeated neuropsychological testing about 3 weeks after ECT found objective as well as subjective memory difficulties to be present, but the authors cautioned that subjective post-ECT reports of memory problems may be influenced also by problems of awareness, questioning their reliability.^10^ Perhaps if routine standard cognitive assessment were available in ECT services, patients could be reassured that in addition to their own ‘report back’ opportunities to identify their concerns about memory problems with their doctor, their cognitive functions (including memory) would be closely monitored for objective change.

## Remaining questions

Although current research appears to indicate that ECT in most patients does not appear to have lasting adverse effects on memory, we can, of course, not be entirely sure that this is always the case for all patients. For example, prospective studies that look at ECT effects on cognition, where baseline neuropsychological testing including consideration of premorbid intellectual ability is included and compared with a control group, could help to further advance our understanding of the cognitive neuroscience surrounding this topic. However, of particular relevance to Vann Jones and McCollum's interesting review, to help address patients' concerns expressed through their subjective reports of ongoing memory difficulties, the following could possibly be helpful to think about. Future research in the area may wish to consider more closely which specific non-cognitive factors, including self-awareness, might account for a discrepancy between actual and reported cognitive impairment. For example, a recent study of patients with acquired brain injury found that most of the variance between actual and perceived cognitive impairment was explained by affective factors such as anxiety or low mood.^11^ Conversely, it may be helpful to also look more closely at which specific symptom(s) of MDD improve post-ECT. As an example, one interesting hypothesis to test would be to determine how much of improvement in depression can be accounted for (or not) by a specific improvement in patients' ability to think or concentrate post-ECT.

As regards clinical practice, one of the possible implications from Vann Jones and McCollum's study is that there may be a need to consider if it is necessary to determine recommended standards of cognitive testing or clearer guidance on testing for UK ECT services. It may, of course transpire, that routine standardised cognitive testing of all patients receiving ECT would possibly be unhelpful or even harmful, raising anxiety-provoking questions in patients' minds that were not there before. However, if standardised testing were deemed necessary to implement, close consideration might need to be given to factors such as which cognitive functions are most relevant to test in the ECT situation, the length of such assessments and, closely related to the latter, what type of assessment (bedside, formal neuropsychological testing or a hybrid approach), among other questions. To conclude, several recent papers illustrate the essence of some of these questions. Although the Montreal Cognitive Assessment^12^ is considered to be useful in monitoring cognitive function after ECT,^13^ on the other hand, cognitive assessment after ECT is also presently thought to not be comprehensive enough, especially if limited to bedside testing only.^9^ As with many of the questions surrounding the clinical practice and neuroscience of ECT, this is not an easy one to provide a definitive answer to.

## About the author

**Dr Rudi Coetzer** is a Consultant Neuropsychologist and Head of Service with the North Wales Brain Injury Service, Betsi Cadwaladr University Health Board NHS Wales, UK, and a Senior Lecturer in Clinical Neuropsychology in the School of Psychology at Bangor University, Wales, UK.
